# Mechanical and Microstructural Performance of Concrete Incorporating Waste Tire Rubber and Recycled Steel Fibers Under Elevated Temperatures

**DOI:** 10.3390/polym18141681

**Published:** 2026-07-08

**Authors:** Ersin Ayhan, Mehmet Kadri Değer, Murat Doğruyol

**Affiliations:** 1 Department of Civil Engineering, Siirt University, Siirt 56000, Türkiye; ersinayhan@siirt.edu.tr; 2 Department of Civil Engineering, Şırnak University, Şırnak 73000, Türkiye; mkadrideger@sirnak.edu.tr

**Keywords:** waste tire rubber, recycled steel fibers, elevated temperature performance microstructural analysis, sustainable concrete, residual compressive strength

## Abstract

This study investigates the thermo-mechanical and microstructural performance of concrete incorporating waste tire rubber (WR) and recycled steel fibers (WS) under elevated temperatures. Four mixtures were prepared: plain concrete (PL), rubber-modified concrete (WR5), and hybrid mixtures containing 0.4% and 0.8% steel fibers (WS0.4WR5 and WS0.8WR5). Specimens were exposed to temperatures of 400 °C, 600 °C, and 800 °C to simulate fire conditions. The results indicate that the incorporation of rubber reduces compressive strength at ambient temperature due to its lower stiffness and weak interfacial bonding. However, the addition of recycled steel fibers significantly improves crack resistance and enhances thermal stability. At 400 °C, the WS0.8WR5 mixture showed a retention rate of 92.9% (absolute strength: 44.32 MPa), compared to 72.2% for plain concrete (absolute strength: 44.11 MPa). Although the hybrid mixture has a lower ambient strength (47.68 MPa vs. 61.07 MPa), its superior retention makes it competitive in fire scenarios. Ultrasonic pulse velocity (UPV) measurements revealed a strong correlation with compressive strength degradation, confirming its effectiveness as a non-destructive indicator of internal damage. Microstructural analyses (SEM, XRD, and TGA-DTA) demonstrated that elevated temperatures lead to dehydration, phase transformation, and increased porosity, while steel fibers help maintain matrix integrity through crack-bridging mechanisms. The findings highlight a synergistic interaction between waste rubber and steel fibers, offering a sustainable and effective approach for improving the fire resistance of concrete.

## 1. Introduction

In modern civil engineering, the integration of sustainable materials and the mitigation of environmental impacts represent pivotal research frontiers. Utilizing waste by-products in construction not only enhances resource efficiency but also addresses the global crisis of end-of-life vehicle tires. With approximately one billion tires reaching their service life annually, their low biodegradability poses a severe environmental challenge [1,2,3]. Consequently, international directives, such as those from the European Tyre and Rubber Manufacturers’ Association (ETRMA), increasingly incentivize the repurposing of these wastes into value-added construction materials [4].

Incorporating waste tire rubber (WR) and recycled steel fibers (WS) into concrete matrices offers a dual advantage. While WS enhances mechanical properties, specifically tensile strength, toughness, and crack resistance, WR improves energy dissipation and performance under dynamic loading, which is particularly beneficial for seismic applications [5]. However, while the ambient-temperature performance of such modified concretes is well-documented, their behavior under extreme conditions, such as fire, requires rigorous investigation.

High-temperature exposure (up to 1000 °C) triggers complex physio-chemical changes in concrete, including C-S-H phase dehydration, thermal incompatibility between aggregates and paste, and internal pore pressure build-up, often leading to explosive spalling [6,7]. Kizilkanat et al. reported that the thermal conductivity of concrete decreased significantly at 600 °C, while the moisture resistance factor already dropped at 300 °C [8]. However, their study did not include mechanical or microstructural analysis. Aksoylu et al. [9] demonstrated that recycled steel wires from waste tires (1–3% by weight) increased compressive strength by up to 46.4% and splitting tensile strength by up to 36.7% at ambient temperature, but their study did not address elevated temperature exposure. The present study extends this knowledge by integrating mechanical, UPV, and microstructural analyses up to 800 °C using waste-derived materials. A critical phenomenon in this context is the “sacrificial” role of rubber particles. At temperatures exceeding 200 °C, rubber begins to decompose, creating an interconnected network of micro-channels [10,11]. These channels serve as pressure relief pathways for water vapor, potentially mitigating the internal stresses that cause spalling—a role similar to that of polypropylene fibers.

Despite these benefits, the degradation of rubber at high temperatures can significantly weaken the concrete’s skeletal integrity. This is where the hybrid use of recycled steel fibers becomes essential. Steel fibers act through a “crack-bridging” mechanism [12], maintaining the structural cohesion of the matrix even after the rubber phase has deteriorated [13]. The synergistic interaction between the thermal relief provided by WR and the mechanical bridging offered by WS represents a promising strategy for developing fire-resistant sustainable concrete [14].

Recent advances in 3D-printed functionally graded concrete plates [15] and FRP-reinforced UHPC composites with hybrid fibers [16] have demonstrated the potential of layered and multi-material systems for enhanced flexural performance and thermal stability. Compared to these advanced manufacturing techniques, the present study investigates a low-cost, waste-derived hybrid system (waste tire rubber + recycled steel fibers) using conventional casting, focusing on fire resistance up to 800 °C.

Although concrete is inherently more fire-resistant than steel or timber, the inclusion of recycled polymers and fibers alters its thermal response. Previous studies indicate that while WR may reduce initial compressive strength, it can potentially mitigate pore pressure by creating sacrificial channels upon melting. Conversely, WS acts through a crack-bridging mechanism, maintaining structural integrity at elevated temperatures [17,18,19].

Previous studies, including the comprehensive review by Thomas and Gupta [20], have focused exclusively on rubberized concrete without considering the synergistic effect of recycled steel fibers. In contrast, the present study introduces a hybrid WR + WS system to enhance crack bridging and thermal stability under fire scenarios.

Despite the growing body of research on rubberized concrete and steel fiber-reinforced concrete, the coupled thermo-mechanical behavior of hybrid systems incorporating waste tire rubber and recycled steel fibers recovered from end-of-life tires remains insufficiently understood. Existing studies have generally focused on either the mechanical performance at ambient conditions or the isolated effects of rubber and steel fibers under elevated temperatures. Furthermore, limited attention has been given to establishing direct correlations between residual mechanical properties, non-destructive ultrasonic measurements, and microstructural evolution after fire exposure.

To address this gap, the present study investigates the synergistic interaction between waste tire rubber and recycled steel fibers in concrete exposed to temperatures up to 800 °C. Unlike previous studies, a comprehensive multi-scale approach combining compressive strength, ultrasonic pulse velocity (UPV), scanning electron microscopy (SEM), energy dispersive X-ray spectroscopy (EDX), X-ray diffraction analysis (XRD), thermogravimetric analysis (TGA) and differential thermal analysis (DTA) were employed to elucidate the governing degradation mechanisms. The novelty of this work lies in establishing a direct relationship between thermo-mechanical deterioration and microstructural evolution in hybrid recycled-material concrete, thereby providing new insights into the development of sustainable and fire-resistant cementitious composites.

## 2. Materials and Methods

### 2.1. Constituent Materials

#### 2.1.1. Cement

CEM I 42.5 N type Portland cement was used in the experimental study. The physical and chemical properties of cement are given in Table 1.

#### 2.1.2. Aggregates

Materials in the ranges of 0–4.00 mm, 4.00–11.20 mm and 11.20–22.40 mm were used as aggregate. The density of sand consisting of fine aggregate is 2.62 g/cm^3^, medium aggregate is 2.69 g/cm^3^, and coarse aggregate is 2.72 g/cm^3^. Aggregates consist of natural stream aggregates. Concrete mixtures were made based on the TS802 [21] standard. Since waste steel fiber-added concrete was produced within the scope of the study, care was taken to ensure that the largest aggregate diameter was 22.40 mm, according to the fiber content selected within the scope of the TS10514 [22] standard.

#### 2.1.3. Waste Tire Fiber Steel (WS)

The diameters of steel fibers separated by mechanical methods from waste tires of heavy vehicles were determined by measurements with the help of an electronic caliper. Accordingly, it was determined that there were two groups of steel fibers with different diameters. The lengths of steel fibers varied due to mechanical disintegration, so their lengths are given as measurement ranges. Their slenderness was determined by dividing their length by their diameter. The physical properties of steel fibers are given in Table 2 [19].

#### 2.1.4. Waste Tire Rubber (WR)

**Figure 1:**
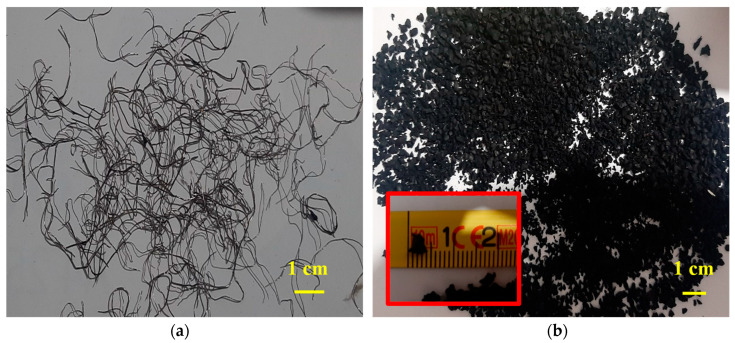
Materials obtained from end-of-life vehicle tires by mechanical separation: (**a**) waste tire steel fibers, (**b**) waste rubber particles of 0–4 mm size [24].

The waste tire rubbers (0–4 mm) replaced 5% of the fine aggregate by volume. However, the technical challenges of incorporating waste rubber into concrete, including poor interfacial bonding and stiffness incompatibility, have been well documented [23]. The waste rubbers were obtained from end-of-life heavy vehicle truck tires through a mechanical separation process, resulting in fragmented particles. The waste rubber particles and steel fibers used in the study are shown in Figure 1.

### 2.2. Mix Proportioning

As shown in Table 3, four concrete mixtures were designed: PL, WR5, WS0.4WR5, and WS0.8WR5. The rubber content was limited to 5% to avoid excessive interfacial degradation [23,25], and the volumetric replacement ratios of fine aggregate with steel fibers were restricted to 0.4–0.8% to prevent the strength loss associated with fiber clumping and workability issues at higher fiber contents [26,27,28]. These proportions enable a systematic evaluation of the WR + WS synergy under elevated temperatures.

The Portland cement content was kept constant at 350 kg/m^3^ for all mixtures, with a water-to-cement ratio of 0.50. Natural aggregates were graded into three size fractions: 0–4 mm (fine aggregate), 4–12 mm, and 12–22 mm (coarse aggregates). In the PL, the fine aggregate content was 813.75 kg/m^3^. For the WR5 mixture, waste rubber (0–4 mm particle size) was added at 13.51 kg/m^3^, replacing an equivalent volume of fine aggregate, thereby reducing the fine aggregate content to 773.06 kg/m^3^. In the hybrid mixtures, waste steel fibers were introduced at 28.36 kg/m^3^ for WS0.4WR5 and 56.72 kg/m^3^ for WS0.8WR5, with a corresponding further reduction in fine aggregate content to 762.58 and 752.10 kg/m^3^, respectively, to maintain constant total volume. The coarse aggregate fractions (4–12 mm and 12–22 mm) remained unchanged across all mixtures at 556.99 and 469.34 kg/m^3^, respectively. A superplasticizer was added at 6.00 kg/m^3^ for all mixtures to maintain workability. The superplasticizer used was a PCE-based admixture (Chryso Delta 8709, Chryso Group, Saint-Gobain, France) with a density of ≈1.06 g/cm^3^. The total masses of the mixtures were 2371.08, 2343.90, 2361.78, and 2379.66 kg/m^3^ for PL, WR5, WS0.4WR5, and WS0.8WR5, respectively, reflecting the different densities of the constituent materials.

#### 2.2.1. Specimen Preparation and Curing

Three 150 × 150 × 150 mm^3^ cubic concrete specimens from each design group were produced according to TS 802 [21] and cured for 28 days at 20 ± 5 °C and 97 ± 2% humidity in a standard controlled atmosphere. The steps involved in making the concrete mixture were as follows: the mixer was filled with coarse aggregates, sand, cement, and steel fibers. After that, they were mixed for three minutes in the mixer before super-plasticizer and water were added. After that, the mixer was run for a further two minutes to create fresh concrete with equally distributed steel fibers [29].

#### 2.2.2. Elevated Temperature Exposure

**Figure 2:**
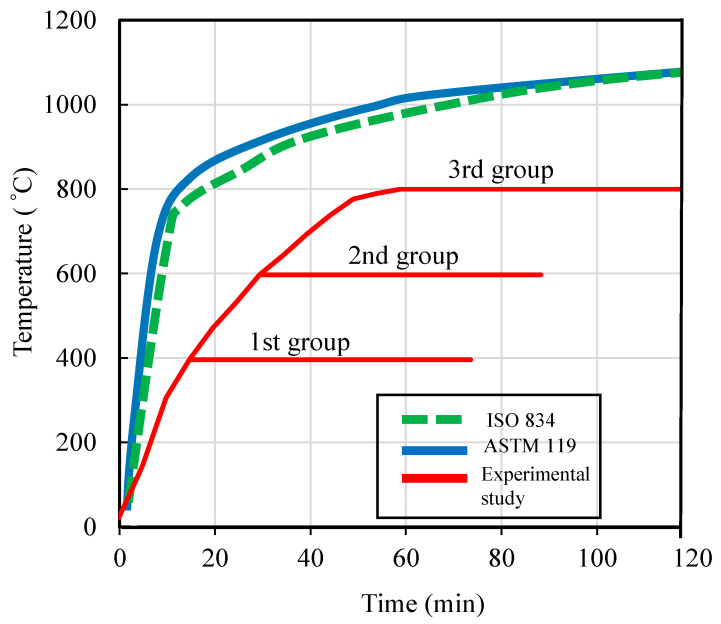
Time–temperature curve of the furnace used in the experimental study compared with ISO 834 and ASTM 119 standard curve [19].

According to ISO 834 [7] and ASTM E119 [30], building materials may reach temperatures above 900 °C within 60 min, depending on the fire scenario. While some studies in the literature have employed longer exposure durations, such as 2 h [31], a 60-min exposure period was adopted in this study based on the ISO 834 standard fire curve. The plain and composite concretes were exposed to fire in an electric muffle furnace for 60 min each, divided into three groups, at temperatures of 400 °C, 600 °C, and 800 °C, analogous to the fire scenario depicted in the standards (Figure 2).

After completing the 60-min exposure at each target temperature, the electric muffle furnace was turned off, and the furnace door was slightly opened (approximately 10 mm) to allow gradual cooling of the specimens in still air. Specimens were left inside the furnace until they reached room temperature (≈25 °C). The cooling duration varied between 8 and 10 h, depending on the maximum exposure temperature. No water quenching or forced air cooling was applied to avoid thermal shock and additional cracking. Once cooled, the specimens were removed for visual inspection, mass loss measurement, and subsequent mechanical and microstructural testing.

#### 2.2.3. Test Method

**Figure 3:**
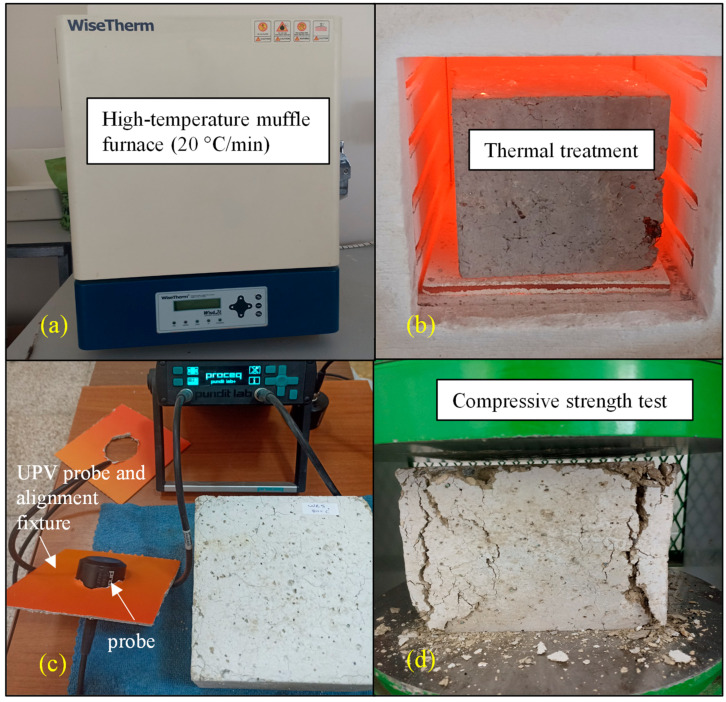
Schematic of the experimental setup for combined thermos-mechanical loading: (**a**) high temperature muffle furnace; (**b**) thermal treatment; (**c**) UPV measurement; (**d**) compressive stress.

Concrete specimens subjected to elevated temperatures were fractured in a press machine, and their compressive strength was assessed. The overall experimental work is outlined in Figure 3. The designs exposed to the fire scenario were compared with the results of the designs at ambient temperature (25 °C). Subsequent to the mechanical test, the crushed concrete specimens were converted into configurations appropriate for microanalysis, and the mechanical outcomes were juxtaposed with SEM, EDX, XRD and, TGA-DTA analyses.

#### 2.2.4. Compressive Strength Test

Three specimens from each design group were tested using a YKM-CM 205-1 concrete testing machine (Yüksel Kaya Makina, Ankara, Turkey) with a capacity of 2000 kN, which complies with TS EN 12390-4 [32] and ASTM C39 [33] standards. The loading rate was maintained constant at 0.3 MPa/s for all tests, in accordance with the standard requirements. The average compressive strengths were then determined from the three specimens.

#### 2.2.5. Ultrasonic Pulse Velocity Test

UPV measurements were performed on cube specimens using a Pundit+ lab device (Proceq, Schwerzenbach, Switzerland) in accordance with ASTM C-597-02 [34]. The transducers had a frequency of 54 kHz, and petroleum jelly (Vaseline) was used as the coupling medium to ensure proper acoustic contact. To ensure accurate direct through-transmission measurements, a custom alignment fixture was employed (shown in Figure 3c). This fixture holds the transmitter and receiver probes coaxially with their active faces exactly opposite each other, eliminating lateral or angular misalignment and thus improving measurement repeatability. For each mixture, three specimens were tested.

#### 2.2.6. Mass Loss Measurement After Elevated Temperature Exposure

To evaluate the deterioration behavior of concrete specimens subjected to elevated temperatures, the mass loss ratio of the 150 mm cube specimens was experimentally determined before and after thermal exposure. Prior to heating, all specimens were dried under laboratory conditions and their initial masses (*M*_1_) were measured using a precision digital balance. After exposure to the target temperatures and subsequent cooling to ambient temperature, the final masses (*M*_2_) of the specimens were recorded again.

The mass loss ratio (Δ*M*) was used as an indicator of moisture evaporation, dehydration of hydration products, and thermally induced internal deterioration occurring within the concrete matrix. The percentage mass loss was calculated according to the following equation:

(1) $$\Delta M=\frac{M_{1}-M_{2}}{M_{1}}\times 100$$

where *M*_1_ is the initial mass of the specimen before elevated temperature exposure (g), *M*_2_ is the final mass of the specimen after cooling (g), and Δ*M* is the mass loss ratio (%).

#### 2.2.7. Microstructural Analysis

The specimens used for microstructural analysis were taken from a depth of 15–30 mm from the surface of each 150 mm cube concrete specimen. The determination of this depth range was based on the temperature gradient occurring in the specimens gradually heated up to 800 °C. The selected depth range of 15–30 mm reflects the homogeneous distribution of the thermal effect within the matrix, enabling reliable analysis of the decomposition of hydration products and the formation of new phases. To maximize the comparability of results and experimental consistency across different mixtures, sampling from all specimen groups was carried out from the same depth range.

Initially, the specimens were prepared for operation on the SEM apparatus. To capture images of the prepared specimens, the surfaces intended for imaging were rendered conductive using the Au coating apparatus. The specimens were positioned in the chamber of the SEM apparatus, after which the process commenced. The surface morphologies of the specimens were analyzed using SEM (Hitachi SU3500 (Hitachi High-Tech Corporation, Tokyo, Japan)) at many scales.

XRD investigations were performed at a wavelength of 1.5406 (λ) within the range of 10 to 90° with a step increment of 0.02° and a scanning speed of 2° per minute.

TGA and DTA were conducted in a nitrogen environment at a heating rate of 10 °C/min utilizing Shimadzu DTG60 AH (Shimadzu DSC 60 A (Shimadzu Corporation, Kyoto, Japan)) thermal analyzers. A sample size of 19 to 22 mg was utilized [19].

## 3. Results and Discussion

The mechanical, physical, and microstructural characterization data (compressive strength, UPV, SEM, XRD, and TGA-DTA) of the plain concrete (PL) specimens used as the reference mixture in this study were previously reported by our research group [19]. Therefore, the following subsections focus primarily on the comparative performance of the waste-modified mixtures under elevated temperatures, while PL data are included as a baseline for reference.

This section compares and discusses the impacts of increased temperature, waste steel fiber, and waste rubber on the visual examination of concrete surfaces, compressive behavior, mass losses, and data collected from characterization analysis tests.

### 3.1. Visual Inspection of Concrete Specimens

**Figure 4:**
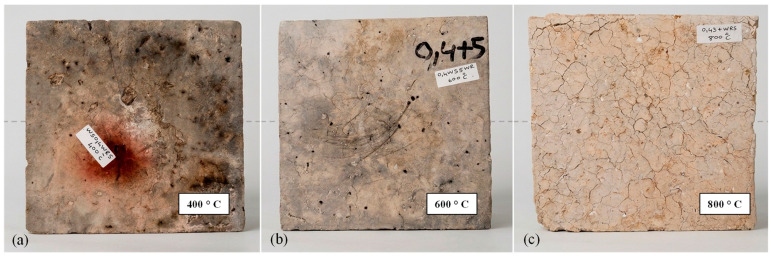
Surface degradation and color change of WS0.4WR5 concrete specimens at elevated temperatures: (**a**) 400 °C; (**b**) 600 °C; (**c**) 800 °C.

The WS0.4WR5 sample specimens illustrated in Figure 4 exhibit surface cracks and color alterations in concrete mixtures following exposure to extreme temperatures. No notable degradation was perceptible to the naked eye in the specimens exposed to the fire scenario up to 400 °C. At 600 °C, capillary fissures and delamination at the peripheries were noted on the concrete surfaces.

At 800 °C, surface fissures became apparent, and edge delamination intensified. The concrete surface, initially light grey at ambient temperature, transformed to pink to red, grey-brown, and grey-white following exposure to 400 °C, 600 °C, and 800 °C, respectively. These color changes are consistent with the findings of Yüzer et al. [35], who reported that such transitions correlate strongly with compressive strength loss in cement-based materials exposed to elevated temperatures. The alteration in color of the specimens was demonstrated to correlate with the chemical and physical transformations undergone by the concrete upon exposure to elevated temperatures [29,36,37,38].

### 3.2. Effect of Elevated Temperatures on Compressive Strength

**Figure 5:**
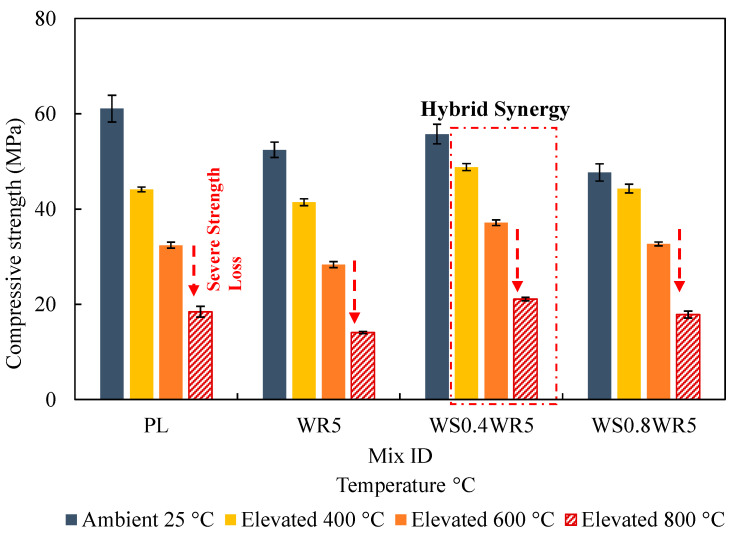
Compressive strength degradation under thermal exposure. Dashed arrows explicitly emphasize the severe strength loss zone triggered at the highest temperature level (800 °C).

**Figure 6:**
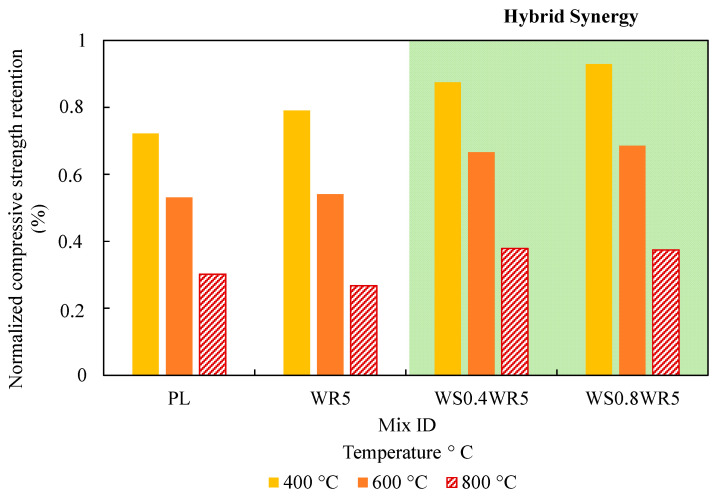
Normalized compressive strength retention (%) of plain and waste-modified concretes after exposure to 400 °C, 600 °C, and 800 °C, relative to ambient (25 °C) values.

Figure 5 presents the compressive strength values of plain and waste-modified concrete mixtures at ambient temperature and after exposure to 400 °C, 600 °C, and 800 °C. Figure 6 presents the normalized compressive strength retention of all concrete mixtures after exposure to elevated temperatures, relative to their ambient values. At ambient temperature, the compressive strengths of PL, WR5, WS0.4WR5, and WS0.8WR5 were 61.07 MPa, 52.43 MPa, 55.75 MPa, and 47.68 MPa, respectively. The reduction in strength for rubber-containing mixtures at 25 °C is consistent with previous studies [3,13], which attribute this behavior to the weak interfacial transition zone between rubber particles and the cement matrix, as well as the lower stiffness of rubber compared to natural aggregates. This interpretation is further supported by our SEM analysis (Figure 10), which reveals visible gaps and poor adhesion around rubber particles at the microstructural level.

With increasing temperature, all mixtures showed progressive strength losses. According to the literature, rubber degradation begins to be appreciable around 200 °C, above which significant yields of volatiles are released. Generally, natural rubber (NR) decomposes first at approximately 380 °C, followed by styrene butadiene rubber (SBR) at 450 °C and butadiene rubber (BR) at 460 °C [39]. Below 200 °C, the meso structure of rubber-modified concrete does not change significantly, and performance degradation remains non-obvious [40]. In the present study, although measurements were not taken at 200 °C, the relatively moderate strength losses observed at 400 °C compared to higher temperatures are consistent with this threshold.

After heating to 400 °C, the meso structure of rubber-modified concrete changes significantly, leading to the formation of many pores in the cement paste, which can release internal vapor pressure [40]. In this study, after exposure to 400 °C, the strength retentions for PL, WR5, WS0.4WR5, and WS0.8WR5 were 72.2%, 79.1%, 87.5%, and 92.9%, respectively, corresponding to compressive strengths of 44.11 MPa, 41.44 MPa, 48.79 MPa, and 44.32 MPa. Notably, the hybrid mixtures containing waste steel fibers exhibited significantly better thermal stability than plain concrete at this temperature, owing to the bridging effect of fibers that restrained thermally induced microcracking [41].

At 600 °C, rubber particles and hydrates decompose completely, resulting in a loosening of the internal structure and a significant deterioration of mechanical performance [40]. In agreement with this, the present study found that after heating to 600 °C, the compressive strengths of PL, WR5, WS0.4WR5, and WS0.8WR5 were 32.43 MPa, 28.34 MPa, 37.13 MPa, and 32.70 MPa, respectively, corresponding to strength retentions of 53.1%, 54.1%, 66.6%, and 68.6%. The superior performance of the hybrid mixtures became more evident at this stage, with WS0.4WR5 maintaining the highest absolute strength. The rubber-only mixture showed slightly lower strength than PL at 600 °C, and its performance declined more sharply at 800 °C due to complete rubber decomposition.

After exposure to 800 °C, all mixtures experienced severe degradation. The compressive strengths of PL, WR5, WS0.4WR5, and WS0.8WR5 fell to 18.45 MPa, 14.06 MPa, 21.10 MPa, and 17.84 MPa, respectively, corresponding to strength retentions of 30.2%, 26.8%, 37.8%, and 37.4%. The hybrid mixtures, especially WS0.4WR5, consistently outperformed both plain and rubber-only concrete at all elevated temperatures, demonstrating that the combined use of waste rubber and waste steel fibers mitigates thermal damage up to 800 °C. However, the substantial strength losses beyond 600 °C highlight the vulnerability of all mixtures to extreme thermal exposure.

Consistent with these findings, previous studies have reported similar temperature-dependent strength degradation patterns in conventional and fiber-reinforced concretes. According to Xiao and König [42], when the temperature exceeded 400 °C, the compressive strength of standard concrete began to decline precipitously, and by 800 °C, almost 80% of its strength was compromised. Similarly, Chan et al. [43] identified the temperature range of 400–800 °C as critical for strength reduction. Savva et al. [44] noted that only a minimal proportion of the original strength persisted in all concrete samples subjected to temperatures above 600 °C, varying from 7% to 25% across all mixtures.

These mechanical test results demonstrate that the incorporation of waste steel fibers improves durability under high temperature exposure [29,45,46]. A similar trend was observed by Unverdi et al. [47] in high-volume fly ash mortars reinforced with industrial steel fibers, where the hybrid composite retained nearly 60% of its late-age compressive strength (32.00 MPa) after exposure to 600 °C, compared to only 33.1% retention in the plain matrix. This confirms that the crack bridging effect of steel fibers, whether recycled or industrial, is a key mechanism for thermal stability. Direct comparison with recent studies further validates our findings. Yang et al. [48] reported that waste steel fiber-reinforced concrete (C40, 1% fiber) retained 44.5% of its compressive strength at 800 °C, which is consistent with our WS0.4WR5 mixture (37.8% retention). Alsaif and Abbas [49] observed that recycled tire steel fibers (RTSF-1.0) achieved a 9.14% strength increase at 300 °C but suffered a 10.14% decline at 500 °C due to fiber–matrix debonding. These results align with our finding that optimal fiber dosage (0.4–0.8%) is critical for balancing ambient strength and thermal resistance. Collectively, these comparisons validate the effectiveness of recycled steel fibers in mitigating thermal degradation of concrete. As shown in Figure 6, the normalized compressive strength retention ratios of all mixtures following exposure to high temperatures indicate that the hybrid mixtures consistently exhibit higher retention ratios, particularly at 400 °C and 600 °C, which further supports the conclusion that recycled steel fibres enhance the thermal stability of rubber-modified concrete up to 600 °C.

Consistently, Liang et al. [50] reported that steel fiber-reinforced rubber concrete with 1.2% steel fiber and 5% rubber substitution exhibited the best post-fire mechanical performance, with compressive, tensile, and flexural strength improvements of 0.23–8.48%, 22.92–44.23%, and 3.03–19.81%, respectively, compared to normal concrete. These findings align with our observation that the WS0.8WR5 mixture (0.8% steel fiber + 5% rubber) showed superior strength retention (92.9% at 400 °C).

### 3.3. Effect of Elevated Temperatures on UPV

**Figure 7:**
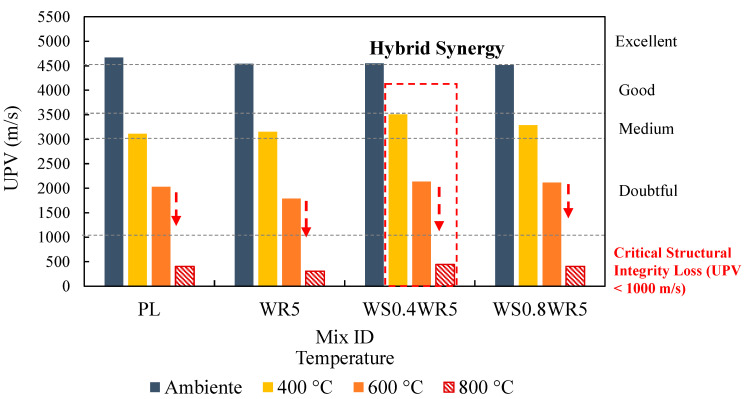
Effect of thermal degradation on concrete UPV. Dashed arrows explicitly indicate the drop into the Critical Structural Integrity Loss threshold across all tested mixes at the highest temperature level.

UPV measurements were conducted on PL and waste-modified concrete specimens at ambient temperature and after exposure to elevated temperatures of 400 °C, 600 °C, and 800 °C. The results, along with the concrete quality classification according to BIS 13311-92-Part I [51], are presented in Figure 7.

As illustrated in Figure 7, all concrete mixtures exhibited UPV values exceeding 4000 m/s at ambient conditions, which corresponds to the ‘excellent’ quality class, indicating a dense and well-compacted internal structure. However, a systematic and marked decrease in UPV values was observed in all specimens as the exposure temperature increased. This degradation is attributed to thermally induced micro-cracks, pore enlargement, and the dehydration of calcium silicate hydrate (C-S-H) gels, all of which disrupt internal continuity and reduce wave transmission efficiency. A dramatic collapse in UPV values was recorded at 800 °C, where all specimens dropped below the 1000 m/s threshold. As highlighted by the ‘Critical Structural Integrity Loss’ zone in Figure 7, these extremely low velocities (averaging 400 m/s) correlate with the formation of macro-scale cracks and the total decomposition of the cementitious matrix, signaling a near-complete loss of structural soundness.

**Figure 8:**
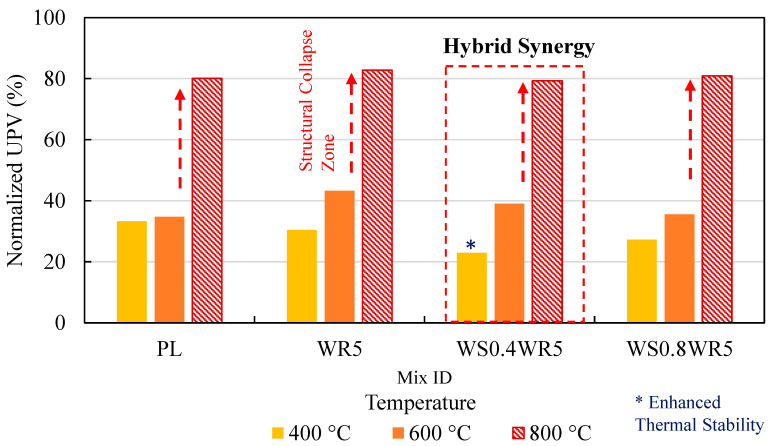
Normalized UPV performance under different temperature exposures. Dashed arrows explicitly track the UPV behavior leading into and within the designated Structural Collapse Zone.

To evaluate the thermal degradation more accurately, the UPV results were normalized by expressing the residual pulse velocity as a ratio of the initial velocity measured at 25 °C. As illustrated in Figure 8, this normalization clearly highlights the relative loss of internal continuity. At 400 °C, the UPV loss ratios for PL, WR5, WS0.4WR5, and WS0.8WR5 were 33.32%, 30.47%, 22.88%, and 27.26%, respectively. These results indicate that hybrid mixtures, particularly WS0.4WR5, exhibit superior resistance to thermally induced damage at moderate temperatures. This behavior is attributed to the crack-bridging effect of the recycled steel fibers, which maintains the continuity of the internal matrix and facilitates wave transmission. The improved UPV retention in fiber-containing mixtures can be explained by the ‘acoustic highway’ effect proposed by Unverdi et al. (2026) [47]. They demonstrated that well-anchored steel fibers provide a continuous solid pathway for ultrasonic waves, even when the surrounding matrix is micro-cracked, thereby reducing UPV loss. In the present study, recycled steel fibers similarly preserved internal continuity, especially at 400 °C and 600 °C, delaying the transition to the critical structural integrity loss zone.

At 600 °C, the degradation became more pronounced, with losses reaching 34.77% for PL and a peak of 43.32% for the WR5 mixture. The significantly higher loss in WR5 confirms that the thermal decomposition of rubber particles increases internal porosity and discontinuities. In contrast, the hybrid mixtures (WS0.8WR5 at 35.56%) partially compensated for this deterioration by limiting crack propagation. However, beyond 600 °C, a drastic collapse was observed, eventually reaching the Critical Structural Integrity Loss zone at 800 °C, where all mixtures suffered losses exceeding 79% and relative velocities dropped below 25% of their original values. This severe degradation correlates with the reduction in compressive strength [52] and signifies a near-total loss of structural soundness.

Interestingly, while the incorporation of waste steel fibers slightly decreased the initial UPV at 25 °C due to reduced homogeneity and increased initial void content [53], these fibers significantly enhanced crack resistance at elevated temperatures compared to the PL group. In plain concrete, unhindered crack growth led to higher void ratios and lower density. Consequently, the addition of recycled steel fibers was demonstrated to be a critical factor in maintaining the internal integrity of rubberized concrete under extreme thermal exposure.

**Figure 9:**
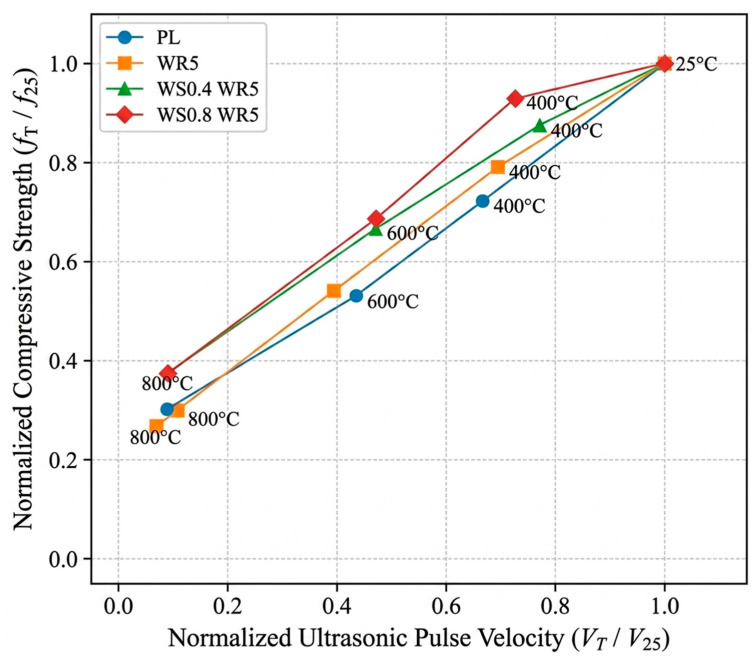
Normalized compressive strength versus normalized UPV of the investigated mixtures under high-temperature effects.

To quantitatively link microstructural damage to mechanical degradation, linear regression analysis was performed between normalized ultrasonic pulse velocity ($V_{T}/V_{25}$) and normalized compressive strength ($f_{T}/f_{25}$) for all mixtures at 25 °C, 400 °C, 600 °C, and 800 °C. As shown in Figure 9, a strong linear correlation was obtained (R^2^ = 0.89), expressed as $f_{T}/f_{25}=0.79\times \left(V_{T}/V_{25}\right)+0.14$. This relationship confirms that UPV loss quantitatively reflects internal crack propagation and porosity increase, enabling non-destructive estimation of residual strength after fire exposure. A direct linear relationship was also established between percentage loss in UPV and percentage loss in compressive strength (R^2^ = 0.89), where each 1% reduction in UPV corresponds to approximately 0.85% reduction in compressive strength, with a baseline offset of 9.6%. This provides a practical tool for post-fire strength assessment using only non-destructive UPV measurements.

### 3.4. SEM Analysis

**Figure 10:**
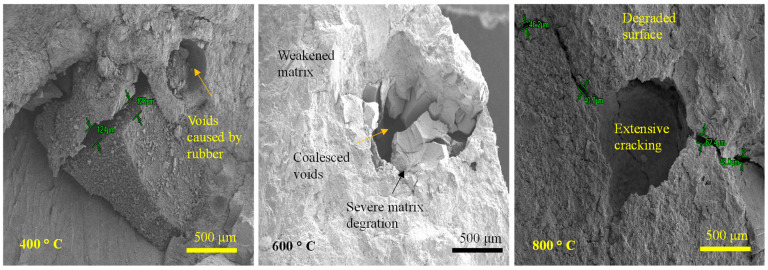
SEM images of rubber substituted specimens (WR5) with temperature increase.

Figure 10 clearly shows that increasing temperature causes microstructural changes on the surfaces of waste-rubber-modified concrete. At 400 °C, distinct voids and microcracks are present, and cracks with widths of 124–128 μm are particularly noticeable. At this temperature, the porous structure caused by rubber incorporation becomes even more pronounced due to the thermal effect. According to Liang et al. [50], rubber particles begin to melt and volatilize at elevated temperatures, leaving behind pore channels that release internal vapor pressure and prevent explosive spalling. This mechanism explains the increased porosity observed in our WR5 samples at 400 °C and above. When the temperature reaches 600 °C, both microcracks and voids grow larger and begin to merge, indicating a weakening of the structural integrity. At 800 °C, there is a significant increase in the size and number of voids and cracks, with crack widths ranging from 40 to 62 μm. At this stage, it is evident that the addition of rubber causes severe degradation and increased porosity at high temperatures, resulting in greater discontinuities on the surface.

**Figure 11:**
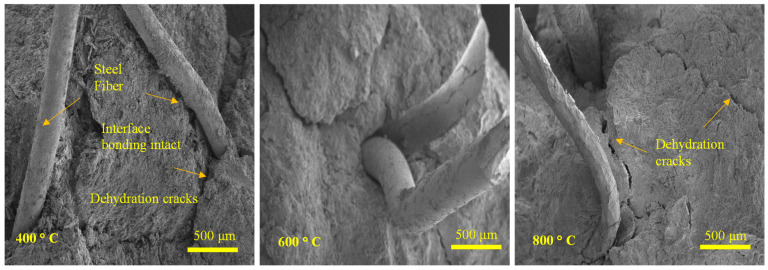
SEM images of fiber steel substituted specimens with temperature increase.

Figure 11 demonstrates how steel fiber addition influences the microstructure of concrete at elevated temperatures. At 400 °C, steel fibers remain embedded within the matrix, and the microstructure of the concrete appears relatively intact. Although minor microcracks and small voids are present around the fibers, no significant segregation is observed. At 600 °C, slight interfacial separation between the fibers and the matrix begins, but the presence of the fibers inhibits crack propagation and helps maintain microstructural strength. At 800 °C, while there is some loss of matrix integrity, the steel fibers continue to limit crack widening, and the porosity remains lower than that observed in the WR5 sample.

According to SEM analyses, void and crack formation increases rapidly with temperature in waste rubber-modified concrete, whereas fiber-reinforced concretes maintain a more compact and coherent structure at high temperatures due to the bridging and crack-limiting effects of the fibers. These findings clearly demonstrate the effectiveness of steel fibers in enhancing high-temperature resistance and microstructural stability.

The start and spread of cracks brought on by high temperatures in concrete were postponed by the bridging and bonding action of steel fibers [54].

The crack-bridging mechanism of steel fibers at elevated temperatures observed in this study (Figure 11) is also emphasized by Akbulut et al. [55], who demonstrated that steel fibers form strong adherence bonds with the matrix and effectively limit the transformation of micro-cracks into macro-cracks under thermal loading.

### 3.5. Energy Dispersive X-Ray (EDX) Microanalysis

**Figure 12:**
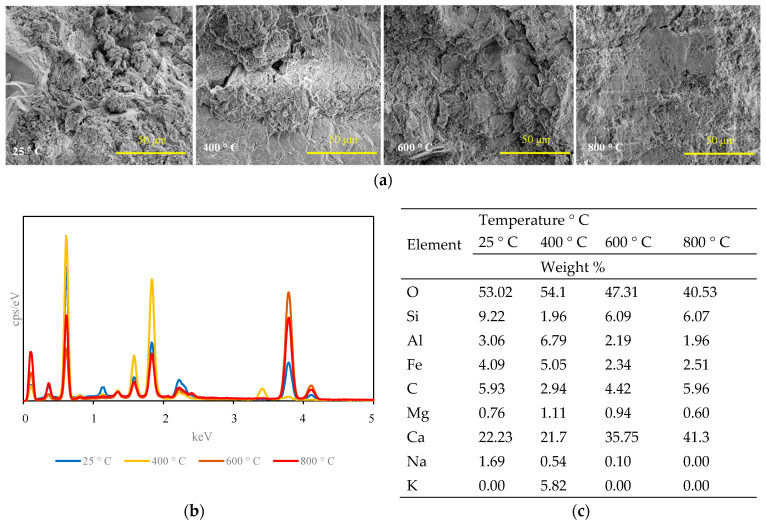
(**a**) SEM images of PL sample; (**b**) EDX curve of PL sample; (**c**) elemental variation as a function of temperature.

**Figure 13:**
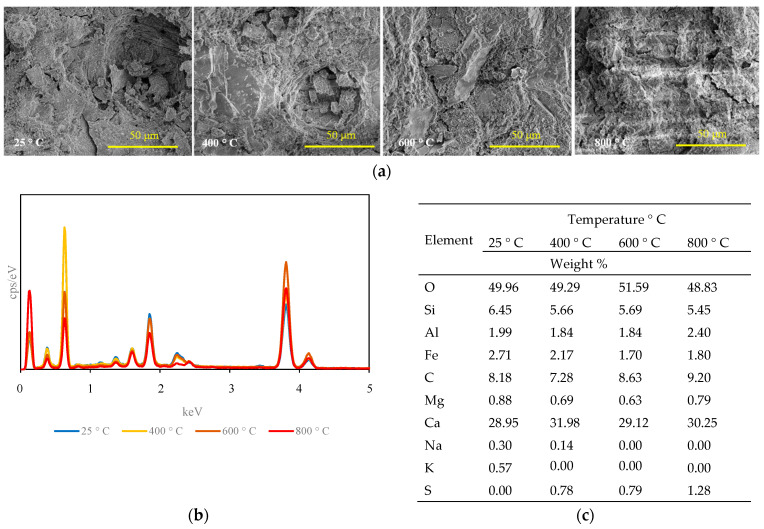
(**a**) SEM images of WR5 sample; (**b**) EDX curve of WR5 sample; (**c**) elemental variation as a function of temperature.

EDX microanalysis was performed to evaluate the elemental composition of the PL and rubber-modified concrete at ambient temperature and after exposure to high temperatures. The results are presented in Figure 12 for the PL sample and in Figure 13 for the WR5 sample.

EDX microanalysis data demonstrate the variations in elemental composition at distinct temperatures between the PL and the sample with WR5. The acquired data offer significant insights into the chemical stability of the binding phase and the phase transitions induced by temperature variations. EDX analysis of the PL sample revealed that oxygen, silicon, and calcium dominate at all temperatures, consistent with the presence of C-S-H and portlandite in cementitious systems. The rise in Si and Al ratios from 25 °C to 400 °C reflects densification of hydration products and microstructural compaction. Nevertheless, once the temperature is above 600 °C, a substantial reduction in the Si ratio and a notable increase in the Ca ratio have been noted. This alteration can be elucidated by the disintegration of C-S-H gel into more stable calcium-based phases and the development of calcium enrichment at elevated temperatures. Likewise, the reduction in the oxygen ratio signifies that dehydration processes are advancing [56]. In the WR5 sample, the elemental distribution exhibits a distinct trend. The reduced Si and Al ratios in this sample, relative to the PL sample, suggest a weakened continuity of the binding phase and a constrained production of C-S-H. The elevated carbon (C) content at all temperature settings is directly attributable to the rubber addition. The noted elevation in the carbon ratio corresponding to the temperature increase can be ascribed to the thermal breakdown and carbonization of the rubber particles.

The restricted fluctuation in the Ca ratio relative to the PL sample suggests that considerable recrystallization or phase condensation did not occur in the WR5 sample at elevated temperatures. Upon assessing the overarching trend of elemental alterations, it is evident that the PL sample saw more significant chemical transformations with rising temperatures. In contrast, the WR5 sample displayed a more erratic and unstable elemental distribution. Notably, after 400 °C, the elemental variations detected in the WR5 sample suggest that the degradation of the rubber phase has increased heterogeneity within the system. This indicates that the rubber additive adversely impacts the continuity of the binder phase at elevated temperatures, thereby diminishing chemical stability. The increasing sulfur (S) content in the WR5 sample with rising temperature is attributed to the thermal decomposition of vulcanization agents originally present in the waste tire rubber [57,58]. In conclusion, the EDX microanalysis results indicate that the control sample demonstrates a more consistent and systematic chemical transformation at elevated temperatures, while the rubber-modified sample reveals a more erratic structure, particularly at high temperatures. These findings suggest that although rubber additives offer specific benefits at low temperatures, they compromise the chemical and microstructural integrity of the material at elevated temperatures [57,58,59]. Consequently, the WR5 mixture exhibited a less uniform elemental distribution compared with the control mixture, which may contribute to its reduced thermo-mechanical performance.

### 3.6. XRD Analysis

**Figure 14:**
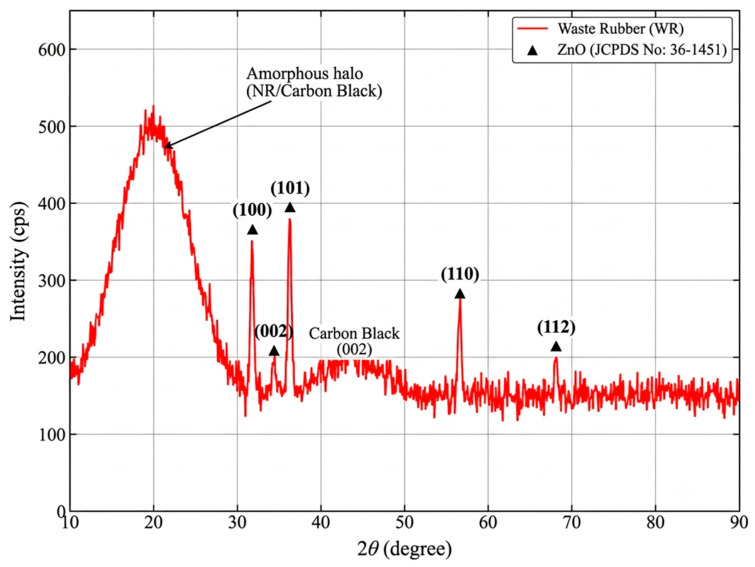
X-ray diffraction (XRD) pattern of the waste rubber (WR) powder.

The XRD pattern of the waste rubber (WR) in Figure 14 confirms its distinct behavior within concrete matrices subjected to elevated temperatures. The dominant amorphous halo observed at 2θ ≈ 15–25° represents the elastomeric phase [60], which is highly susceptible to thermal degradation, typically initiating above 200 °C. When integrated into the concrete matrix, the thermal decomposition of this amorphous phase induces the formation of interconnected micro-channels. These pathways effectively release internal water vapor pressure, thereby mitigating the risk of explosive thermal spalling. Conversely, the sharp diffraction peaks at 31.8° and 36.3° correspond to zinc oxide (ZnO), indicating a highly stable crystalline structure that remains intact at elevated temperatures and functions as thermally stable inorganic micro-fillers. The broad and irregular profile of the diffraction pattern further verifies that the waste tire rubber possesses a high degree of amorphousness with non-predominant crystalline phases. Characterizing this structure is crucial for understanding the behavior of rubber as a mineral additive and evaluating its potential microstructural interactions within the concrete matrix. In a similar vein, Hakamy (2021) [61] emphasized that incorporating additives into the cementitious matrix, particularly nano-silica phases, significantly promotes the formation of new mineral phases, thereby enhancing the overall structural stability of the matrix.

**Figure 15:**
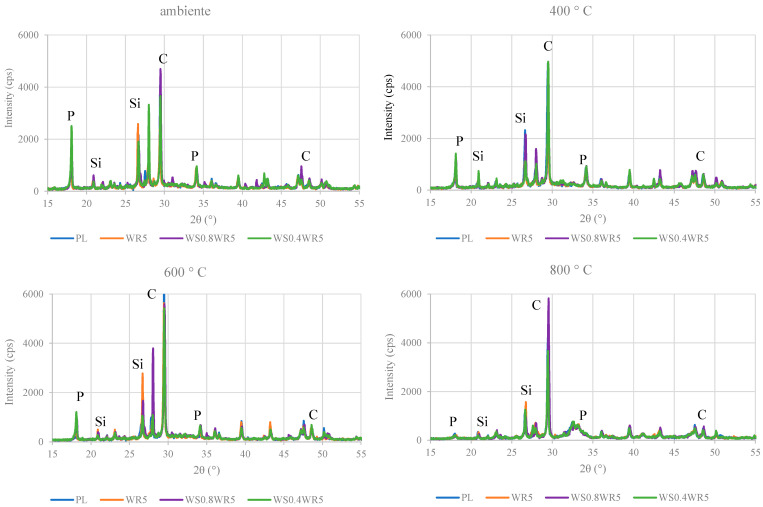
According to XRD analysis, the change in the components of the samples depending on the temperature. Portlandite (P), SiO_2_ (Si), and calcite (CaCO_3_).

The XRD patterns presented in Figure 15 reveal the temperature-dependent evolution of mineral phases in the investigated concrete mixtures. A clear reduction in peak intensity and the number of identifiable crystalline phases is observed as the exposure temperature increases, indicating progressive degradation of hydration products and structural reorganization within the cement matrix.

At ambient temperature, the diffraction patterns are characterized by well-defined and sharp peaks corresponding to crystalline hydration products such as CH, C–S–H, quartz (SiO_2_), and CaCO_3_. These findings are consistent with typical hydrated cement systems and confirm the presence of a stable and well-developed microstructure. In agreement with the literature, the relatively high crystallinity at this stage reflects the formation of well-structured mineral phases within the matrix [61].

At 400 °C, a slight decrease in peak intensity is observed, indicating the onset of thermal decomposition processes. This behavior can be attributed to the gradual loss of physically bound water and the initial destabilization of hydration products. The observed changes are consistent with previous findings, which report that early-stage thermal exposure leads to partial dehydration and microstructural rearrangement without complete phase destruction [62].

At 600 °C, a significant reduction in the intensity of CH peaks is evident, indicating the decomposition of calcium hydroxide into CaO and water vapor. This transformation is widely reported in the literature and represents a critical threshold for the degradation of cementitious materials [63]. Simultaneously, the breakdown of the C–S–H gel structure leads to a marked reduction in the overall crystallinity of the matrix. The decrease in peak intensity and partial disappearance of characteristic peaks confirm severe microstructural deterioration at this temperature level.

At 800 °C, the XRD patterns show a substantial disappearance of hydration-related peaks, accompanied by the emergence of broader and less intense diffraction signals. This indicates a transition toward more thermodynamically stable phases, primarily CaO, along with increased amorphization of the matrix. The decomposition of CaCO_3_, as reflected by the reduction in its characteristic peak intensity around 29.3° 2θ, further supports the occurrence of advanced thermal degradation. Similar observations have been reported in previous studies, where elevated temperatures led to significant changes in crystallinity and phase composition [64].

In addition, the variation in SiO_2_ peak behavior, as discussed in Figure 15, suggests that silica phases remain relatively stable up to moderate temperatures but begin to exhibit peak broadening and intensity reduction at higher temperatures. This behavior is associated with structural rearrangement and partial amorphization, which is consistent with findings reported by Oufakir and Khouchaf (2023) [65] and other studies highlighting the thermal sensitivity of silica-based phases [66].

Furthermore, the presence of waste rubber influences the XRD patterns by promoting a more amorphous structure, as also indicated in Figure 15. The broad and diffuse peaks observed in the 20–30° 2θ range confirm the predominantly amorphous nature of rubber-derived components, which is consistent with the findings of Hakamy (2021) [61]. At elevated temperatures, the decomposition of rubber contributes to increased porosity and matrix discontinuity, which indirectly accelerates the degradation of crystalline phases.

Overall, the XRD results clearly demonstrate that increasing temperature leads to (i) progressive decomposition of hydration products (CH and C–S–H), (ii) transformation of phases into thermally stable compounds such as CaO, and (iii) a significant reduction in crystallinity accompanied by increased amorphization. These findings are in strong agreement with the literature and provide a mineralogical explanation for the mechanical strength losses and microstructural deterioration observed at elevated temperatures.

### 3.7. Thermogravimetric and Differential Thermal Analysis (TGA and DTA)

Free water in the samples persists up to 100 °C, while capillary water, gel water, and chemically bound water in C-S-H and Gypsum dehydration are lost between 100 and 250 °C [67]. Capillary and physically absorbed water constitute the predominant mass of the cement paste, whereas the majority of C-S-H evaporates at temperatures exceeding 250 °C. The conversion of CH at 450 °C results in considerable mass loss, as Portlandite undergoes decomposition into free lime by dehydration between 450 and 550 °C [68,69,70,71]. In TGA, the decomposition of CaCO_3_ is characterized by a distinct mass loss step corresponding to the release of CO_2_. While this thermal decarbonation occurs across a broad temperature interval that can vary depending on the nature of the carbonate and experimental conditions, it is typically observed in the range of approximately 550–800 °C, with the onset temperature often situated above 650 °C [72,73].

**Figure 16:**
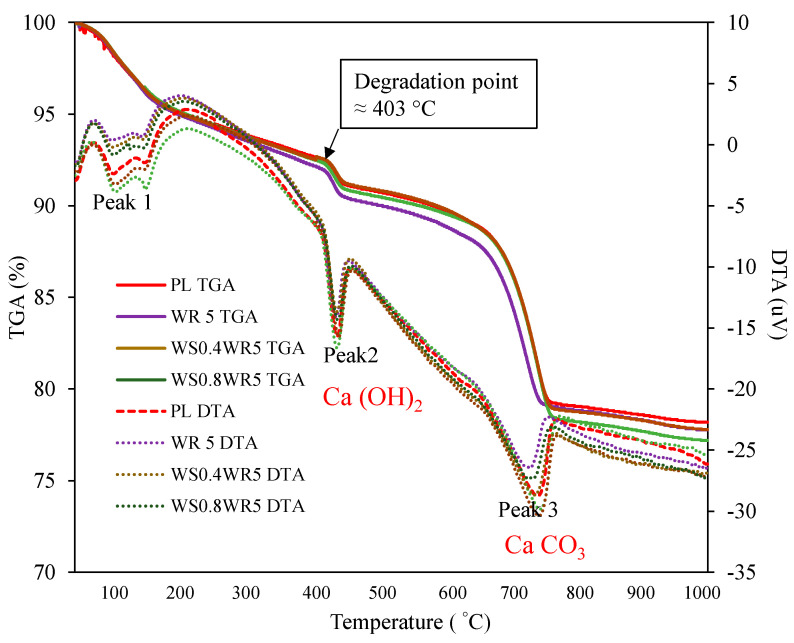
TGA curves of powdered samples over the temperature range of 0–1000 °C.

**Figure 17:**
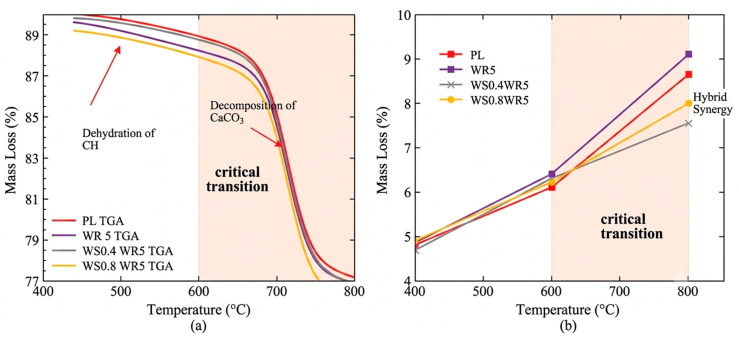
Validation of mass loss trends: (**a**) TGA analysis highlighting the decomposition of hydration products and carbonated phases, and (**b**) experimentally measured mass loss (%) of 150 mm cube specimens. The consistency between both methods validates the thermal degradation phases of the rubberized-fiber composite.

The thermogravimetric analysis of the concrete powders is presented comparatively in Figure 16, while Figure 17 provides a comparative analysis of the mass loss percentages obtained from TGA of these powders and from direct scale measurements of 150 mm concrete cubes after exposure to elevated temperature.

Figure 16 presents the TGA curves of powdered concrete samples over the temperature range of 0–1000 °C. No significant differences in total mass loss were observed among the mixtures, indicating comparable initial hydration and free water contents. The TGA results identified an inflection point at approximately 403 °C, corresponding to the onset of portlandite (CH) decomposition. This temperature range also coincides with the beginning of significant reductions in compressive strength, UPV, and mass retention, suggesting that temperatures above 400 °C represent a critical transition zone for thermo-mechanical degradation. Accelerated mass loss observed beyond 600 °C further confirms the progression of this deterioration process (Figure 17) [47,74].

Beyond this transition zone, progressive dehydration of the C–S–H gel and decomposition of CH accelerate, leading to increased porosity, microcrack initiation and propagation, and a gradual loss of matrix cohesion. These microstructural changes are consistent with the reductions in compressive strength and ultrasonic pulse velocity observed throughout the experimental program, confirming the close relationship between chemical degradation and thermo-mechanical performance.

From an engineering perspective, exposure to temperatures above approximately 400 °C may initiate irreversible deterioration within the cementitious matrix, whereas temperatures in the range of 500–600 °C represent a critical stage at which substantial losses in load-bearing capacity and structural integrity are generally observed [75,76]. Furthermore, the residual strength of concrete after cooling is typically lower than its original ambient-temperature strength [77]. Consequently, concrete elements exposed to such temperatures during fire events should be considered potentially compromised and subjected to detailed post-fire assessment before being returned to service [78].

As demonstrated in Figure 17, a consistent trend is observed between the micro-scale thermal analysis of the cementitious matrix and the macro-scale weight loss of the cubic specimens. However, it is important to distinguish the underlying mechanisms: while the TGA profiles (a) specifically highlight the chemical decomposition of hydration products within the cement paste, the physical weight loss measurements (b) reflect the cumulative mass reduction from both the matrix dehydration and the volatilization of the waste rubber particles. The accelerated divergence in weight loss beyond 600 °C in the cubic specimens compared to the matrix-only TGA results confirms that the thermal degradation of rubber significantly contributes to the overall increase in porosity. This synergistic mass loss process explains the severe structural integrity failure and the dramatic decline in UPV values previously observed at the 800 °C threshold. The mass loss trends observed in this study are consistent with previous findings. Abdi Moghadam and Izadifard [79] reported that weight loss of plain concrete increased with temperature, reaching 10.78% at 800 °C, while the inclusion of steel fibers reduced mass loss at all tested temperatures. Similarly, in our study, the hybrid mixtures (WS0.4WR5 and WS0.8WR5) exhibited lower mass loss compared to plain concrete, particularly at 800 °C, confirming that steel fibers help maintain matrix integrity by reducing thermally induced spalling and cracking.

Notably, in the hybrid mixtures containing both steel fibers and rubber, the mass loss was lower than that of the control specimen, supporting the synergistic interaction between steel fibers and rubber. These findings indicate that the combined use of fibers and rubber can significantly improve the thermal stability and high-temperature resistance of the cement matrix. The inclusion of steel fibers contributes to maintaining matrix integrity by inhibiting microcrack formation and propagation, thereby enhancing the retention of mechanical properties after exposure to elevated temperatures. These findings confirm that the thermomechanical performance of fibre-reinforced concrete is determined not only by the presence of fibres, but also by their thermal stability, interfacial compatibility and the interaction with developing microstructural damage mechanisms [74,80].

## 4. Conclusions

This study investigated the thermo-mechanical and microstructural performance of concrete incorporating waste tire rubber (WR) and recycled steel fibers (WS) under elevated temperatures up to 800 °C. Based on the experimental findings, the following conclusions can be drawn:

1. The incorporation of 5% waste tire rubber reduced the ambient compressive strength of concrete due to the lower stiffness of rubber particles and the weak interfacial transition zone formed between rubber and the cementitious matrix.
2. The inclusion of recycled steel fibers partially compensated for the strength reduction caused by rubber incorporation by enhancing crack-bridging capacity and improving matrix integrity. Among the investigated mixtures, the hybrid composites exhibited superior thermal resistance compared with plain and rubber-only concretes.
3. Elevated temperature exposure progressively deteriorated all concrete mixtures. However, the hybrid mixtures demonstrated significantly higher residual strength retention. The WS0.8WR5 mixture retained approximately 92.9% of its original compressive strength after exposure to 400 °C, while the WS0.4WR5 mixture exhibited the highest residual strength at 600 °C and 800 °C.
4. UPV measurements revealed a strong correlation with compressive strength degradation (R^2^ = 0.89), indicating that ultrasonic testing can be effectively employed as a reliable non-destructive technique for post-fire assessment of concrete structures.
5. SEM observations confirmed that increasing temperature induced microcracking, pore enlargement, and matrix deterioration. In contrast, recycled steel fibers effectively limited crack propagation through a crack-bridging mechanism, preserving microstructural continuity.
6. XRD and TGA-DTA analyses demonstrated progressive decomposition of hydration products, particularly portlandite and C-S-H phases, above 400 °C. Significant phase transformation and increased amorphization were observed beyond 600 °C, corresponding closely with the measured reductions in compressive strength and UPV.
7. The synergistic interaction between sacrificial rubber decomposition and fiber-induced crack bridging contributed to improved thermal stability. While rubber decomposition generated pressure-relief channels that may reduce internal vapor pressure, steel fibers maintained structural coherence during thermal degradation.
8. From an engineering perspective, the combined utilization of waste tire rubber and recycled steel fibers offers a sustainable approach for producing fire-resistant concrete while simultaneously promoting the valorization of end-of-life tire waste.
9. The findings provide new multi-scale evidence linking mechanical performance, ultrasonic response, and microstructural evolution of hybrid recycled-material concrete under fire conditions. Future studies should investigate long-term durability, spalling resistance, and structural-scale behavior under realistic fire scenarios to further validate the practical applicability of these sustainable composites.

## Figures and Tables

**Table 1:** The properties of cement.

SiO_2_	Al_2_O_3_	Fe_2_O_3_	CaO	MgO	SO_3_	Na_2_O	K_2_O	Loi *	Blaine (m^2^/kg)	Density (g/cm^3^)
21.12	4.62	3.80	62.94	2.73	2.20	0.02	0.35	2.22	338	3.10

* Loi = Loss on ignition.

**Table 2:** Properties of steel fiber.

Group of Steel Fiber	Diameter (mm)	Length (mm)	Fragility	Weight Ratio
1st Group	0.11	25–55	227–500	61%
2nd Group	0.29	35–50	120–172	39%

**Table 3:** Concrete mixture quantities (kg/m^3^).

Mix Proportions	OPC	Water	Rubber	Steel Fiber	Aggregate		
					(0–4 mm)	(4–12 mm)	(12–22 mm)
PL	350	175	0	0	813.75	556.99	469.34
WR5	350	175	13.51	0	773.06	556.99	469.34
WS0.4WR5	350	175	13.51	28.36	762.58	556.99	469.34
WS0.8WR5	350	175	13.51	56.72	752.10	556.99	469.34

## Data Availability

Data are contained within the article. Further inquiries can be directed to the corresponding author upon reasonable request.
